# Retraction: The Arctic charr (*Salvelinus alpinus*) genome and transcriptome assembly

**DOI:** 10.1371/journal.pone.0247083

**Published:** 2021-02-09

**Authors:** Kris A. Christensen, Eric B. Rondeau, David R. Minkley, Jong S. Leong, Cameron M. Nugent, Roy G. Danzmann, Moira M. Ferguson, Agnieszka Stadnik, Robert H. Devlin, Robin Muzzerall, Michael Edwards, William S. Davidson, Ben F. Koop

After this article [1] was published, further analysis revealed that the sample used for the reported genome analysis was more likely from the closely related species, Dolly Varden (*Salvelinus malma*), or may have been from a hybrid between the two species.

We stand by the genome and transcriptome assembly as scientifically valid and representative of the sample analyzed. However, the identity of that individual is now in question and consequently the article’s title, results, and conclusions attributing the sequences to Arctic charr are not upheld.

In light of this issue, we retract the article. We have also requested that NCBI change the species name on the genome sequence deposition to indicate that the data were derived from unclassified species in the *Salvelinus* genus, or *Salvelinus sp*.

The authors apologize for the issues with this article.

AS and RM either could not be reached or did not respond to correspondence about this retraction. The other authors all agreed with the retraction decision.
